# Abstracts

**DOI:** 10.1038/sj.bjc.6601481

**Published:** 2003-12-10

These abstracts were presented as part of the ‘Iressa’ Clinical Experience (ICE) meeting held in Madrid in June 2003 and are reproduced with the authors’ permission. Data from several of these abstracts have subsequently been published (full references are shown in the journal articles in which the ICE abstracts are cited) and further publications are expected.

## Antitumour effect of eighth-line gefitinib (‘Iressa’, ZD1839) in a heavily pretreated patient with adenocarcinoma of unknown primary origin (probably pulmonary)

**A Awada^1^ and J Klastersky^1^**

*^1^Institut Jules Bordet, Brussels, Belgium*

This case report describes the objective response of a 70-year-old, heavily pretreated male smoker with adenocarcinoma of unknown primary origin (probably pulmonary) following eighth-line gefitinib (‘Iressa’, ZD1839) therapy. In February 1996 he presented with adenocarcinoma, and over the next 5 years received seven lines of chemotherapy (gemcitabine; ifosfamide plus a platinum-based agent; paclitaxel; 5-fluorouracil; docetaxel plus farnesyltransferase inhibitor; vinorelbine; and doxorubicin plus temozolomide) along with radiotherapy for brain metastases. Objective responses were seen when docetaxel plus a farnesyltransferase inhibitor and vinorelbine were used, and stable disease was observed following treatment with paclitaxel. In April 2001, echography and CT scans found metastases in the cervical lymph nodes (LN), liver and mediastinum, and lactate dehydrogenase (LDH) and carcinoembryonic antigen (CEA) levels were 258 U L^−1^ (normally 120–140 U L^−1^) and 22.3 *μ*g L^−1^ (normally 0–2.5 μg L^−1^), respectively. At this time, the patient began taking 250 mg day^−1^ gefitinib orally, as part of the ‘Iressa’ Expanded Access Programme, concomitantly with atenolol. At 67 days after beginning gefitinib therapy, complete responses were observed in the cervical LN and liver metastases, and a partial response was seen in the mediastinum metastases; LDH levels were 143 U L^−1^ and CEA levels were 2.3 *μ*g L^−1^ (confirmed 15 days later). Gefitinib was well tolerated and the only adverse event noted was pruritus. The last contact with the patient was 113 days after starting gefitinib therapy, by which time his quality of life and lifestyle had both improved. However, the patient died suddenly after this, with the cause of death thought to be cardiac in origin.

## Gefitinib (‘Iressa’, ZD1839) as third-line therapy for a patient with symptomatic brain metastases from non-small-cell lung cancer

**M Azémar^1^, C Stoll^1^ and C Unger^1^**

*^1^Tumour Biology Clinic, Freiburg, Germany*

A 57-year-old male smoker was diagnosed with stage IIIb non-small-cell lung cancer (NSCLC) in April 2000 with extended pleural carcinosis of the left lung and exterior pericardial carcinomatosis. Bleomycin plus tetracycline was given initially followed by gemcitabine; a second pleurodesis with tetracycline did not prevent further pleural effusion. Treatment with cisplatin plus gemcitabine (four cycles) and therapy with an Ep-Cam directed antibody, IGN 101, led to disease stabilisation for 18 months. In February 2002, two small brain lesions were detected and treated with radiation (20 Gy each) followed by adjuvant cisplatin plus gemcitabine (three cycles). Oral gefitinib (‘Iressa’, ZD1839) 250 mg day^−1^ was started in June 2002 to consolidate the disease stabilisation. After 1 month, the patient reported neurological symptoms (left hemiparesis and imbalance) and underwent surgery to remove the main lesion. Antiepileptic medication was prescribed after convulsions in August 2002. The patient was hospitalised in November 2002 for neurological symptoms and again in January 2003 for phlebothrombosis and Gram-negative sepsis. Radiological assessment confirmed stabilisation of the primary tumour. The patient reported no adverse reactions to gefitinib, other than the neurological symptoms. In February 2003, gemcitabine and cisplatin were resumed to treat the brain metastases and rising carcinoembryonic antigen levels. However, the patient died 2 weeks later after a generalised seizure. In conclusion, the primary tumour was stabilised by third-line gefitinib therapy for 8 months. The patient's general condition was limited only by neurological symptoms caused by previously irradiated brain metastases. This tumour control in conjunction with surgery for cranial metastases might be of further interest, as the primary tumour did not directly contribute to this patient's death.

## Use of gefitinib (‘Iressa’, ZD1839) in advanced non-small-cell lung cancer for routine clinical practice

**J Bendel^1^, J Häusler^1^, S Korfee^1^, G Antoch^1^, T Gauler^1^ and W Eberhardt^1^**

*^1^University Hospital Essen, Essen, Germany*

From March 2002 to February 2003, 50 patients (34 male, 16 female) received oral gefitinib (‘Iressa’, ZD1839) 250 mg day^−1^ treatment as part of the ‘Iressa’ Expanded Access Programme. At the start of gefitinib treatment, the median age was 60.5 (range 34–78) years and the median Eastern Cooperative Oncology Group performance status was 1 (range 0–2). Most patients in this series had stage IV metastatic disease and approximately 50% had adenocarcinoma. The median number of prior chemotherapy regimens was two (range 0–6), with approximately 90% of pretreated patients having received a platinum-based regimen. Six patients were chemonaive and 31 patients had received previous radiotherapy. The median duration of gefitinib treatment was 12.5 (range 1–46) weeks. In 21 patients a temporary halt of tumour growth, documented by radiological imaging ⩾4 weeks from the start of treatment, has been observed; of these, four patients experienced a partial response, three of whom had demonstrated a response to previous treatment. At the time of writing, for patients demonstrating a response to gefitinib, the median duration of disease control was 18 (range 4–46) weeks and tumour control maintained for ⩾25 weeks was observed in seven patients. Ten patients continue on gefitinib treatment and 36 (72%) patients remain alive. We have observed a trend in symptom improvement and preliminary results from quality-of-life analysis suggest that an improvement of dyspnoea is the most important surrogate marker of an improvement in patients’ well-being. In conclusion, in this series of patients with advanced non-small-cell lung cancer, gefitinib has caused symptom improvement and a prolonged period of tumour control.

## Gefitinib (‘Iressa ’, ZD1839) in patients with pretreated stage IIIb-IV non-small-cell lung cancer entering the ‘Iressa’ Expanded Access Programme at tge University Federico II, Naples

**AR Bianco^1^, V Damianov^1^, E Matano^1^ and R Bianco^1^**

*^1^University Federico II School of Medicine, Naples, Italy*

A total of 82 patients with advanced non-small-cell lung cancer (NSCLC) who had failed previous chemotherapy (at least two lines of chemotherapy including cisplatin and taxanes) entered the ‘Iressa’ Expanded Access Programme at our institution from September 2001 to April 2003. Eight patients had brain metastases (treated with radiotherapy), seven patients had received radiotherapy for bone metastases, seven patients had received radiotherapy for lung metastases and 13 patients had obtained a partial remission following chemotherapy. All patients were given 250 mg day^−1^ gefitinib (‘Iressa’, ZD1839) orally. In all, 65 patients met the eligibility criteria, and 49 have completed ⩾3 months of therapy and are, therefore, evaluable. Patient demography is as follows: male : female, 35 : 14 patients; median age 59 (range 29–80) years; Eastern Cooperative Oncology Group performance status ⩽2; squamous-cell carcinoma, 23 patients; adenocarcinoma (including one patient with bronchioloalveolar), 25 patients; large-cell carcinoma, one patient; and stage IIIb/IV disease, eight out of 41 patients. One female patient with adenocarcinoma (plus brain and lung metastases) has experienced a complete response. After 3 months of therapy, 18 patients had stable disease (SD) and 30 patients had progressive disease (PD). At 6 months, three patients had SD and nine patients had PD, and at 9 months, three patients had SD. After 1 year, two patients (one still receiving gefitinib) had SD. The tolerability of gefitinib was good, with two patients experiencing mild, drug-related skin rash. Although the response rate was low, gefitinib offers definite palliation in pretreated advanced NSCLC, with few side effects.

## Refractory non-small-cell lung cancer: clinical experience with gefitinib (‘Iressa’, ZD1839) in 28 patients who entered the ‘Iressa’ Expanded Access Programme at the Sydney Cancer Centre

**M Boyer^1^**

*^1^Sydney Cancer Centre, Sydney, Australia*

A total of 40 patients with non-small-cell lung cancer (NSCLC) who had received prior chemotherapy or were unsuitable for chemotherapy entered the ‘Iressa’ Expanded Access Programme at the Sydney Cancer Centre from September 2001 to April 2003. Patients who had previously received gefitinib (‘Iressa’, ZD1839) were excluded. Data are presented here for the first 28 patients (19 men, nine women) studied. The most common histology was adenocarcinoma (12 patients), followed by large cell (seven patients), squamous cell (six patients) and bronchioloalveolar (three patients). The majority of patients (17 out of 28, 61%) had an Eastern Cooperative Oncology Group performance status (PS) of 1; two had a PS of 0, five had a PS of two and 2 had a PS of 3 (PS was unknown for 2 patients). Five patients had not received prior chemotherapy. Other patients had received one, two or three chemotherapy regimens (12, seven and four patients, respectively). All patients received gefitinib 250 mg day^−1^. Disease progression was assessed every 8–12 weeks. Four patients had a partial response, three patients had stable disease and 17 patients had progressive disease (four patients were not assessable). The median duration of treatment was 70 days for non-responders and 222 days for responders. The median survival was 154 days. In total, 16 (57%) patients experienced rash, four (14%) reported diarrhoea and two (7%) reported nausea and vomiting. These results indicate that gefitinib may have a role in the treatment of some patients with NSCLC.

## Gefitinib (‘Iressa’, ZD1839) in patients with brain metastases from non-small-cell lung cancer: report of four cases

**F Cappuzzo^1^, A Ardizzoni^2^, H Soto-Parra^3^, C Gridelli^4^, P Maione^4^, M Tisea^2^, C Calandri^1^, S Bartolini^1^, A Santoro^3^ and L Crinò^1^ [a]**

*^1^Bellaria Hospital, Bologna, Italy; ^2^Istituto Nazionale per la Ricerca sul Cancro, Genoa, Italy; ^3^Istituto Clinico Humanita-Rozzano, Milano, Italy; ^4^S.G Moscati Hospital, Avellino, Italy*

Four patients (aged 47–65 years; male : female, 1 : 3) with non-small-cell lung cancer (NSCLC) and brain metastases received gefitinib (‘Iressa’, ZD1839) 250 mg day^−1^ as part of the ‘Iressa’ Expanded Access Programme. All patients had adenocarcinoma. Two patients were non-smokers and two had a prior smoking history. All patients had previously been treated with at least two lines of chemotherapy including at least one platinum-based regimen. In addition, three patients had received prior whole-brain radiotherapy, terminated at least 3 months before starting gefitinib. After 3 months, one patient had a complete response in the brain with stabilisation of extracranial disease, while the other three patients partially responded to gefitinib, both in the brain and extracranial sites. Treatment duration was 3+, 5, 7 and 12+ months. Gefitinib was generally well tolerated, with skin toxicity recorded in two patients (grade 1/2). All patients experienced symptomatic improvement while on gefitinib therapy. The presence of brain metastases has been an exclusion criterion in clinical studies to date. However, this case study suggests that gefitinib is effective in patients with previously treated brain metastases and warrants further investigation.

## Gefitinib (‘Iressa’, ZD1839) in patients with brain metastases from non-small-cell lung cancer: report of four cases

**F Cappuzzo^1^, C Calandri^1^, S Bartolini^1^ and L Crinò^1^ [b]**

*^1^Bellaria Hospital, Bologna, Italy*

Four patients (two male, two female) aged 53–65 years with non-small-cell lung cancer (NSCLC): adenocarcinoma (two patients), squamous-cell carcinoma (one patient) and bronchioloalveolar carcinoma (one patient), developed brain metastases (BM) and extracranial disease. All the patients had previously received first-line platinum-based chemotherapy, and two patients had received whole-brain radiotherapy for BM. Gefitinib (‘Iressa’, ZD1839) 250 mg day^−1^ was used to treat two patients with evidence of brain disease progression and two patients with asymptomatic BM and progressive disease in the extracranial sites. After 3 months of gefitinib, all patients had a partial response in the brain and extracranial sites. However, two patients discontinued gefitinib after 8 and 15 months due to disease progression. To date, two patients remain on gefitinib after 6 and 11 months of treatment. Gefitinib was generally well tolerated, with only grade 1 and grade 2 skin toxicity in two patients and one patient, respectively. While on gefitinib, all patients experienced symptom improvements and a better quality of life. These preliminary findings suggest that gefitinib may be effective in NSCLC patients with pretreated BM and that BM may not be an exclusion criterion for future gefitinib studies. However, large clinical trials are needed to validate these proposals.

## Gefitinib (‘Iressa’, ZD1839) given in the ‘Iressa’ Expanded Access Programme in patients with non-small-cell lung cancer

**A Chioni^1^, F Barbieri^2^, E Baldini^1^, C Orlandini^1^, S Ricci^1^ and PF Conte^2^**

*^1^Ciari University Hospital, Pisa, Italy; ^2^Policlinico University Hospital, Pisa, Italy*

A total of 74 patients with advanced non-small-cell lung cancer (NSCLC), who had failed to respond to one or more previous chemotherapy regimens and were ineligible for further treatment, received gefitinib (‘Iressa’, ZD1839) 250 mg day^−1^ monotherapy as part of the ‘Iressa’ Expanded Access Programme. The demography of this patient group comprised a male : female ratio of 56 : 18, with a median (range) age of 65 (43–81) years. Tumour types included squamous-cell carcinoma (27 patients), adenocarcinoma (18 patients), bronchioloalveolar carcinoma (seven patients), undifferentiated large cells (10 patients) and unspecified NSCLC (12 patients). The median (range) performance status (PS) was 1 (0–3) and 53% of patients had previously failed to respond to a second-line, taxane-containing regimen. Of the randomised patients, 97% were evaluable and no clinical remission was observed. Disease progressed in 47 patients, although disease stabilised in 25 patients. Median progression-free survival and overall survival were 11.6 weeks (range 10 days to 80 weeks) and 18.4 weeks (range 10 days to 86.6 weeks), respectively. Survival rates were found to differ significantly depending on PS prior to treatment (PS 0–1 *vs* PS 2–3; *P*<0.0001). In general, gefitinib was well tolerated, with the most common side effects being grade 1 diarrhoea in 5.4% of patients and grade 1 cutaneous toxicity in 8%. Two patients had grade 3 diarrhoea and one patient had grade 3 diarrhoea plus grade 3 cutaneous toxicity. This case series demonstrates that gefitinib therapy is well tolerated and results in disease stabilisation in 34% of heavily pretreated patients at an advanced stage of disease. In total, 11 patients continued to receive gefitinib treatment.

## Gefitinib (‘Iressa’, ZD1839) in advanced non-small-cell lung cancer patients progressed to chemotherapy

**H Cortes-Funes^1^, M Constenla Figueiras^2^, S Martín-Algarra^3^, P Salinas^4^, B Massutí^5^, P Gascón^6^ and R Rosell^7^**

*^1^Hospital Universitario 12 de Octubre, Madrid, Spain; ^2^Hospital Pontevedra, Pontevedra, Spain; ^3^Clínica Universitaria, Navarra, Spain; ^4^MD Anderson International Cancer Center, Madrid, Spain; ^5^Hospital General, Alicante, Spain; ^6^Hospital Clínico, Barcelona, Spain; ^7^Hospital German Trias i Pujol, Barcelona, Spain*

We have retrospectively assessed the safety profile and antitumour activity of 250 mg day^−1^ gefitinib (‘Iressa’, ZD1839) in 113 pretreated patients with advanced non-small-cell lung cancer (NSCLC) treated as part of the ‘Iressa’ Expanded Access Programme. Patient demography is as follows: male : female 89 : 24; median age 61 (range 36–83) years; stage I-IIa/IIIb-IV, 30/70%; number of tumour sites 1/2/3, 43/27/31%; adenocarcinoma 41%, squamous-cell carcinoma 40%, large-cell carcinoma 15%; number of prior chemotherapy regimens 1/2/⩾3, 21/49/30%; Eastern Cooperative Oncology Group performance status 0–1/2–3/4, 73/25/1%. The median duration of treatment was 81 (range 29–457) days. Ninety-one patients were evaluable for response and three partial responses were observed (3.2%). Disease control rate (response plus stable disease) was 39.8% (95% confidence interval [CI] 29.9, 49.8) and was unaffected by histology, stage at diagnosis or the number of prior chemotherapy regimens. Median time to progression was 3.5 months (95% CI 3.2, 3.8) and median survival was 6.7 months (95% CI 4.7, 8.6). Skin toxicity, the most frequent adverse event, occurred in 42.5% of patients (3.5% grade 3/4). Other toxicities included diarrhoea (21.2%; 0.9% grade 3/4), asthenia (20.4%; 5.3% grade 3/4), nausea and vomiting (10.6%; 0.9% grade 3/4), anorexia (9.7%; 3.5% grade 3/4), neurological toxicity (9.7%; 1.8% grade 3/4) and pulmonary toxicity (0.9%; 0.9% grade 3/4). No gefitinib-related deaths were observed. These data confirm the acceptable safety profile and antitumour activity of gefitinib therapy in pretreated patients with NSCLC. While the response rate was low, a promising rate of disease control has been observed.

## Correlation between epidermal growth factor receptor expression and tumour response in patients with advanced non-small-cell lung cancer treated with gefitinib (‘Iressa’, ZD1839)

**F de Braud^1,2^, C Noberasco^1,2^, G Curigliano^1,2^, M De Pas^1,2^, L Dodaro^1,2^, S Manzoni^1,2^, A Milani^1,2^, A Rocca^1,2^, F Ferrucci^1,2^, G Ferretti^1,2^ and G Pelosi^1,2^**

*^1^European Institute of Oncology, Milan, Italy; and ^2^Regina Elena Cancer Institute, Rome, Italy*

From March 2001 to February 2002, 79 patients with previously treated, locally advanced or metastatic non-small-cell lung cancer (NSCLC) began oral gefitinib (‘Iressa’, ZD1839) 250 mg day^−1^ therapy. Their characteristics were: male/female, 51/28; median (range) age, 56 (31–77) years; adenocarcinoma (51 patients), squamous-cell carcinoma (14 patients), bronchioloalveolar (three patients), undifferentiated (four patients); median (range) number of prior chemotherapy regimens, 2 (1–6). The most common metastatic sites were lung (72 patients), lymph nodes (44 patients), bone (25 patients), liver (18 patients) and brain (11 patients). A total of 14 patients were not assessable (three ceased treatment, seven died and four had not received treatment for a sufficiently long period). Of 65 patients evaluable for response, five (8%) had a partial response (PR) and 19 (29%) had stable disease (SD). Median durations of PR and SD were 7 and 4 months, respectively. Gefitinib was generally well tolerated. The most common adverse events (67 patients) were mild (National Cancer Institute Common Toxicity Criteria grade 1/2) skin reactions (30%), diarrhoea (9%) and nausea (4%). Grade 3/4 skin reactions occurred in 2% of patients. Immunohistochemical detection of epidermal growth factor receptor (EGFR) expression was studied in patients with PR or SD (group A) and nonresponders (group B). EGFR expression (% cells) was mean±s.d. (range) 69.5±27.0 (20–95) in group A (11 patients) and 47.1±43.4 (0–95) in group B (12 patients); *P*=0.241. The experiences of these patients with advanced NSCLC demonstrate gefitinib to be effective for people who have received prior chemotherapy. All responders were EGFR-positive in ⩾20% of cells (mean 69.54%). According to these preliminary immunohistochemical data, EGFR status should be determined.

## Impact of gefitinib (‘Iressa’, ZD1839) in a patient with brain metastasis from non-small-cell lung cancer

**J de la Cruz^1^ and N Giacomi^1^ [a]**

*^1^Centro Oncologico de Excelencia, La Plata, Buenos Aires, Argentina*

In June 2000, a 56-year-old female nonsmoker was diagnosed with a lung adenocarcinoma (stage III, T3N2M0). Thoracic surgery was performed with a right lobectomy. Chemotherapy with paclitaxel+carboplatin (six cycles) and lung radiotherapy achieved a complete response. During follow-up in November 2001, the patient showed loss of strength in her hands and an MRI scan showed a sole image in the right parietal region. Surgery was performed with posterior whole-brain radiotherapy, followed by three cycles of treatment with docetaxel. In December 2001, the patient suffered pleural effusion and, in April 2002, hepatic nodules were revealed by CT scan and hepatic ultrasound. After 1 month of treatment with gefitinib (‘Iressa’, ZD1839) 250 mg day^−1^ the patient achieved a great benefit in symptom relief (70% reduction in pain and dyspnoea), weight increase and a performance status of 1. She was able to return to normal life and analgesic use was drastically reduced. Imaging diagnosis until March 2003 showed stable disease; furthermore, the patient continued to be free from central nervous system (CNS) tumours. In April 2003 the response time was >11 months and the overall survival was >34 months. Gefitinib was well tolerated, with the adverse events comprising skin rash (grade 2) and myalgia (grade 1). Treatment with gefitinib in this patient has demonstrated that a clear benefit in clinical response can be achieved even following heavy pretreatment with cytotoxic agents and radiotherapy. We conclude that it is possible to achieve a long duration of response and improvement of symptoms in patients with CNS metastases treated with gefitinib.

## Prolonged survival and clinical benefits with gefitinib (‘Iressa’, ZD1839) in a patient with brain metastasis from non-small-cell lung cancer

**J de la Cruz^1^ and N Giacomi^1^ [b]**

*^1^Centro Oncologico de Excelencia, La Plata, Buenos Aires, Argentina*

A 56-year-old male patient with a 30-year history of smoking presented with cough and thoracic pain in July 1998. He was diagnosed with locoregional (stage IIIb) epidermoid lung carcinoma (50 × 60-mm lump in the upper right lobule with mediastinal adenopathies), concomitant hypertension and chronic obstructive pulmonary disease. He received treatment as follows: paclitaxel+carboplatin (six cycles); docetaxel (six cycles); and gemcitabine (three cycles)+lung radiotherapy, all of which resulted in partial responses. In July 2001, he presented with instability while walking, an MRI scan showed a sole 2.5-cm metastasis in the cerebellum. Surgery was performed followed by whole-brain radiotherapy and three cycles of gemcitabine treatment. In September 2001, progression at the pulmonary and osseous level was apparent, the patient had a performance status (PS) of 3. Gefitinib (‘Iressa’, ZD1839) 250 mg day^−1^ was administered for 12 months. Imaging diagnosis at 3, 6 and 9 months showed stable disease and he remained free from tumours in the central nervous system (CNS). Improvements in dyspnoea (70%), pain (70%), cough (50%), quality of life (75%), lifestyle (100%) and PS (1) were seen within 30 days. The patient gained weight and returned to full-time work. Tolerability was good, with skin rash (grade 2), myalgia (grade 1) and slight vision impairment. The patient's overall survival was 57 months. This illustrates that gefitinib treatment produces benefits in clinical response even in patients with poor PS who have already been heavily pretreated. Long duration of response and prolonged overall survival are achievable in patients with CNS metastasis, for whom no other treatment is currently available.

## Analysis of the efficacy and tolerability of gefitinib (‘Iressa’, ZD1839) in previously treated patients with non-small-cell lung cancer enrolled in the ‘Iressa’ Expanded Access Programme

**K de Leeuw^1^, D Schallier^1^, E Sermijn^1^, C Fontaine^1^, B Neyns^1^, I Samijn^1^ and J de Grève^1^**

*^1^Oncology Centre AstraZeneca-VUB, Belgium*

In this retrospective analysis, the efficacy and tolerability of gefitinib (‘Iressa’, ZD1839) monotherapy was evaluated in 28 patients with stage IV non-small-cell lung cancer (NSCLC) who had failed previous treatments, including chemotherapy. Patients were treated with oral gefitinib 250 mg day^−1^ for a median duration of 43.5 (range 7–256) days. Gefitinib was given as second-line therapy to seven patients, as third-line therapy to 16 patients and as fourth-line therapy to five patients. Median overall survival was 84.5 (range 7–256) days. Eight patients experienced stable disease for a median duration of 201 (range 59–256+) days. No objective responses were observed. Gefitinib was well tolerated with no grade 3 or 4 toxicities reported. Eight patients developed mild-to-moderate skin rash and eight patients reported mild-to-moderate diarrhoea. In our heavily pretreated patients with NSCLC, gefitinib was well tolerated and produced long-term stable disease in a significant proportion of patients. Treatment with gefitinib should therefore be an option in NSCLC patients who have exhausted, or are too frail to support, known active treatments. Further research into the molecular determinants of response to gefitinib is warranted.

## Efficacy of gefitinib (‘Iressa’, ZD1839) in non-small-cell lung cancer metastatic to the brain

**E Diaz-Cantón^1^**

*^1^Centro de Educacion Medica e Investigaciones Clinicas, Buenos Aires, Argentina*

In January 2000, a 79-year-old female patient with metastatic non-small-cell lung cancer received radiotherapy to bone and brain metastases followed by 3-weekly, first-line, palliative chemotherapy with paclitaxel (200 mg m^−2^) and carboplatin (AUC 6) given on Day 1. The patient experienced severe intolerance to chemotherapy within two cycles. Chemotherapy was discontinued due to toxicity and the patient was included in the ‘Iressa’ Expanded Access Programme on compassionate grounds. After 3 months, a complete response (CR) of short duration (1 month) was documented in the brain. Gefitinib (‘Iressa’, ZD1839) 250 mg day^−1^ was well tolerated with no deterioration in performance status. A mild cutaneous acneiform rash was the only adverse event. The patient died 4 months after beginning gefitinib therapy due to nonspecific alveolitis. While the CR could be explained by the delayed effect of radiotherapy prior to gefitinib treatment, this would be unlikely considering that there were no significant changes in two CT brain scans following brain irradiation in January and March 2002. Additionally, 2 weeks after starting gefitinib, serum alkaline phosphatase levels and pain in her thoracic and lumbar spine increased and a blastic reaction of the spine was documented (CT scan); these could have been related to progressive disease in the bone in the context of a mixed response. In conclusion, gefitinib therapy, which was well tolerated, resulted in a CR in the brain of short duration. Further research is required to better understand the activity of this drug in metastatic disease on the central nervous system.

## A 60-year-old man with metastatic non-small-cell lung cancer at diagnosis survived 20 months

**B Dieriks^1^, D Galdermans^1^, L Bedert^1^, H Slabbynck^1^ and D Coolen^1^**

*^1^Algemeen Ziekenhuis, Middelheim, Belgium*

A 60-year-old man, who had stopped smoking 10 years earlier, was diagnosed with stage IV non-small-cell lung cancer in July 2001. Metastatic sites included lung, liver and brain and his WHO performance status was 0. The patient initially received six cycles of combination therapy with vinorelbine (25 mg m^−2^) and gemcitabine (1000 mg m^−2^), both given on Days 1 and 8 of a 3-weekly cycle (August–December 2001), and demonstrated a partial response. Subsequently, the patient received 4 cycles of docetaxel (75 mg m^−2^ every 3 weeks, April–June 2002) but did not respond to this second-line therapy. At this point, he was in a very bad condition, with dyspnoea and cough due to lung metastasis, and confusion and headache due to brain metastasis. Oral gefitinib (‘Iressa’, ZD1839) 250 mg day^−1^ was given for a period of 6 months (June–December 2002), during which time the patient experienced a partial response with dramatic radiological improvement. A skin reaction was reported, which was treated for 10 days with the antibiotic doxycycline (100 mg day^−1^). Disease-specific symptoms of dyspnoea and cough improved within 10 days of receiving gefitinib and had disappeared after 2 weeks of treatment, and the improvement in the patient's quality of life enabled him to return to his job for more than 6 months. The patient died in February 2003, having spent just the last week of his life in a palliative care unit.

## Clinically meaningful response to the epidermal growth factor receptor tyrosine kinase inhibitor gefitinib (‘Iressa’, ZD1839) in non-small-cell lung cancer

**A Gelibter^1^, A Ceribelli^1^, M Milella^1^, M Mottolese^1^, A Vocaturo^1^ and F Cognetti^1^**

*^1^Regina Elena Cancer Institute, Rome, Italy*

A 39-year-old man, with no history of smoking, presented in June 2000 with poorly differentiated bronchioloalveolar adenocarcinoma with high expression of epidermal growth factor receptor (EGFR) detected immunohistochemically, and amplification of the EGFR gene detected by chromogenic *in situ* hybridisation. Treatment with six cycles of cisplatin and gemcitabine achieved a partial response that lasted 7 months. Carboplatin and paclitaxel were given but after three cycles the disease progressed. Vinorelbine was started and resulted in stable disease lasting 5 months. In October 2001 the patient's Eastern Cooperative Oncology Group performance status (PS) was 2 and he was heavily symptomatic (cough, dyspnoea, fever and severe restrictive pulmonary deficit, which required treatment with corticosteroids and oxygen). A chest CT scan showed metastatic disease. Oral gefitinib (‘Iressa’, ZD1839) 250 mg day^−1^ was started. Within 2 weeks his dyspnoea and fever resolved and by week 4 his cough had abated. After 8 weeks his PS was 0 and by week 12 he required no supportive care. Pulmonary function improved steadily at 7 and 17 weeks and a chest CT scan, performed at 10 weeks, showed almost a complete resolution of the clinical picture. Gefitinib was well tolerated, although a mild acneiform rash was observed at week 2, which spontaneously decreased by Week 7. The patient has received gefitinib for 52 weeks and remission maintained up to 32 weeks. In conclusion, we consider that all patients with advanced or recurrent non-small-cell lung cancer, irrespective of their disease histology or EGFR status, should be considered for inclusion into clinical trials of signal transduction inhibitors.

## A case of diffuse interstitial pneumonitis in a patient with stage IV non-small-cell lung cancer receiving gefitinib (‘Iressa’, ZD1839) treatment

**R Gervais^1^, J Chasles^1^, A Rivière^1^and J-J Michels^1^**

*^1^Centre François Baclesse, Caen, France*

This case report describes the treatment of a female smoker (aged 45 years) who was diagnosed with stage IIIb non-small-cell lung cancer in August 2001. She had no coexisting illnesses and no other lung complications. Initially she was treated with cisplatin and vinorelbine (three cycles), with an outcome of partial response, and this was followed with thoracic radiotherapy. In April 2002 the disease progressed to stage IV, with metastases in the adrenal glands and liver. Second-line docetaxel (six cycles) commenced in October 2002, which initially stabilised the disease but eventually further disease progression was observed. Following these relapses after two chemotherapy regimens and radiotherapy, treatment with oral gefitinib (‘Iressa’, ZD1839) 250 mg day^−1^ was begun. However, a diffuse interstitial pneumonitis occurred in the first 2 weeks of treatment. Despite initial improvement following treatment with corticosteroids, the patient died 10 days after the interstitial pneumonitis was diagnosed. The total duration of gefitinib treatment was 25 days.

## Gefitinib (‘Iressa’, ZD1839) in special patient populations (elderly ⩾70 years or performance status ⩾2) with advanced non-small-cell lung cancer: a case series report from the ‘Iressa’ Expanded Access Programme

**C Gridelli^1^, P Maione^1^, A Rossi^1^, C Guerriero^1^, ML Barzelloni^1^ and C Ferrara^1^ [a]**

*^1^SG Moscati Hospital, Avellino, Italy*

A total of 59 patients with advanced non-small-cell lung cancer (NSCLC) were entered into the ‘Iressa’ Expanded Access Programme and given gefitinib (‘Iressa’, ZD1839) 250 mg day^−1^. Patients were either elderly (⩾70 years old) or had performance status (PS) (Eastern Cooperative Oncology Group) ⩾2. *Elderly group* consisted of 18 patients (17 male, one female), median age 73.5 years, with stage IIIb (three patients) or IV (15 patients) disease and PS 1/2/3 in 4/11/3 patients. The disease histologies were squamous-cell carcinoma (SCC) (10 patients), adenocarcinoma (six patients), bronchioloalveolar carcinoma (one patient) and undefined NSCLC (one patient). Gefitinib was received as first-line (one patient), second-line (seven patients) or ⩾third-line treatment (10 patients). All patients were evaluable. No objective responses were seen and stable disease (SD) was achieved in one patient. The most frequent adverse events were grade 1 and 2 skin changes in four patients and one patient, respectively, and grade 1 diarrhoea in three patients. *Unfit patient group* consisted of 41 patients (26 male, 15 female), median age 60 years, with stage IIIb (four patients) or IV (37 patients) disease and PS 2/3 in 29/12 patients. Their disease histologies were SCC (17 patients), adenocarcinoma (21 patients) and undefined NSCLC (three patients). Patients received gefitinib as first-line (one patient), second-line (16 patients) or third-line (24 patients) treatment. From 39 evaluable patients, two patients with adenocarcinoma reported a partial response, four patients had SD and six patients had controlled disease. The most frequent adverse events were grade 1 diarrhoea in two patients and grade 2 hypertransaminasaemia in one patient. These data suggest that gefitinib is well tolerated in elderly or unfit patients with advanced NSCLC.

## Gefitinib (‘Iressa’, ZD1839) in heavily pretreated non-small-cell lung cancer patients: a case series report from the ‘Iressa’ Expanded Access Programme

**C Gridelli^1^, A Rossi^1^, P Maione^1^, L Musto^1^, F Del Gaizo^1^ and G Airoma^1^ [b]**

*^1^SG Moscati Hospital, Avellino, Italy*

Gefitinib (‘Iressa’, ZD1839) 250 mg day^−1^ was administered as a single agent in the ‘Iressa’ Expanded Access Programme to 83 nonhospitalised patients (aged 33–80 years) with advanced non-small-cell lung cancer (NSCLC). Patients received gefitinib as first-line (two patients), second-line (35 patients) and ⩾third-line (46 patients) treatment. The cohort consisted of 64 male and 19 female patients (median age 61 years) with stage IIIb (11 patients) and stage IV (72 patients) disease, and performance status (Eastern Cooperative Oncology Group) 0/1/2/3 in 4/24/40/15 patients. The disease histologies were adenocarcinoma (39 patients), squamous-cell carcinoma (35 patients), bronchioloalveolar carcinoma (2 patients) and undefined NSCLC (7 patients). To date, 71 patients are evaluable for response and tolerability. Objective responses were reported in four of the evaluable patients (complete response, one male patient; partial response, three female patients). The disease histology of all responders was adenocarcinoma. Epidermal growth factor receptor (EGFR) expression data were available for two responders; both were EGFR negative. Stable disease and disease control were seen in 12 and 16 of the evaluable patients, respectively. The most frequent adverse events were: grade 1 and 2 skin changes (six and two patients, respectively); grade 1 diarrhoea (seven patients); grade 2 hypertransaminasaemia (one patient); and grade 1 epistaxis (one patient). Our data confirm the activity and tolerability of gefitinib in heavily pretreated patients with advanced NSCLC.

## Gefitinib (‘Iressa’, ZD1839), an option for patients with recurrent non-small-cell lung cancer who have failed chemotherapy: a case series report of 92 patients

**A Haringhuizen^1^, HFR Vaessen^1^, P Baas^1^ and N van Zandwijk^1^**

*^1^The Netherlands Cancer Institute, Amsterdam, The Netherlands*

Between May 2001 and September 2002, 92 patients with recurrent, advanced non-small-cell lung cancer (NSCLC) received 250 mg day^−1^ gefitinib (‘Iressa’, ZD1839) in the ‘Iressa’ Expanded Access Programme. Patients were aged 33–76 years; 33% had performance status (WHO) ⩾2; 86% had stage IV disease and 14% had stage III; 62% (57 patients) had adenocarcinoma (including six patients with bronchioloalveolar carcinoma). In all, 62% of patients had received first-line chemotherapy, 30.5% had received >first-line chemotherapy and 7.5% were chemonaive. Median treatment duration was 10.7 (range 0.4–75.3) weeks, although 26 patients continue to receive gefitinib at the time of reporting. Objective responses (8.7%; one complete, seven partial) were observed in patients with adenocarcinoma and the median duration of response was 5.0 (range 1.20–15.8) months. In total, 37% of patients had stable disease, giving an overall disease-control rate of 45.7%. Median survival was 5.4 (range 0.21–18.4) months and 1-year survival approached 10%, although this may change when data are updated. A total of 6.5% of patients had progression-free survival ⩾6 months. Gefitinib was well tolerated and most side effects were mild. The most frequent were grade 1/2 diarrhoea and skin rash, seen in 36.2 and 22.9% of patients, respectively. In all, 26% of patients received palliative radiotherapy during treatment without additional toxicity. One patient had radiological signs of interstitial lung disease (ILD), without symptoms. Gefitinib treatment was maintained and the patient attained a partial response lasting >14 months, with the ILD resolving spontaneously. These data confirm the activity and good tolerability of gefitinib in unselected NSCLC patients who failed previous therapy.

## Gefitinib (‘Iressa’, ZD1839) for patients with advanced non-small-cell lung cancer treated in the ‘Iressa’ Expanded Access Programme at a single institution in Brazil

**A Katz^1^, O Smaletz^1^, SD Simon^1^, GC Rene^1^, PM Hoff^1^ and J Tabacof^1^**

*^1^Hospital Israelita Albert Einstein, Centro Paulista de Oncologia, São Paulo, Brazil*

A total of 26 patients (16 male, 10 female) aged 38–84 years (median 67 years) with non-small-cell lung cancer (NSCLC) (stage IIIb/IV 1/25) received oral gefitinib (‘Iressa’, ZD1839) 250 mg day^−1^ until progression or unacceptable toxicity developed. Their median performance status (Eastern Cooperative Oncology Group) was 1. In all, 25 patients had previously received a platinum and taxane based-regimen, and one patient was chemonaive. The median treatment duration with gefitinib was 50 (range 1–635) days. Three patients who died within 7 days of treatment initiation were not evaluated for response. Of the remaining patients, all of whom received treatment for at least 4 weeks, 19% (95% CI, 4–46%) had a partial response and 52% (95% CI, 25–75%) had disease stabilisation. The median time to progression was 1.7 (range 0.1–21.2) months and median survival was 3.2 (range 1.7–26.2+) months. A significant proportion of patients experienced prolonged clinical benefit: 12 patients survived for more than 6 months, seven patients for more than 9 months and three patients for more than 1 year. Seven patients continue to be treated with gefitinib. The most frequently reported toxicities were grade 1 skin rash (16%) and grade 1/2 diarrhoea (42/11%). No grade 3/4 toxicities were seen. In conclusion, our results with gefitinib in this heavily treated population is comparable with other studies in patients with advanced NSCLC; gefitinib has a significant activity with acceptable toxicity.

## Treatment of non-small-cell lung cancer in the ‘Iressa’ Expanded Access Programme: experience of the medical university of Gdańsk

**A Kowalczyk^1^, R Dziadziuszko^1^, E Szutowicz-Zielinska^1^, A Badzio^1^ and J Jassem^1^**

*^1^Medical University of Gdańsk, Poland*

Between June 2002 and May 2003, 34 patients with stage IIIb–IV, heavily pretreated non-small-cell lung cancer (NSCLC) received gefitinib (‘Iressa’, ZD1839) 250 mg day^−1^ in the ‘Iressa’ Expanded Access Programme. Of these, 32 patients were evaluable for objective and symptomatic response and survival, comprising 24 men (75%) and eight women (25%) (mean age 60 (range 30–79) years). There were 20 cases (63%) of squamous-cell carcinoma, eight (25%) adenocarcinoma and four (12%) other NSCLC. In all, 12 patients (38%) had previously undergone pulmonary resection. In total, 26 patients (81%) had received first-line chemotherapy with a median 4 (range 1–8) cycles. Second-line chemotherapy was administered to 11 patients (34%) and third-line chemotherapy to 1 (3%). Prior radical or postoperative radiotherapy was applied in four patients (13%) and palliative radiotherapy in 16 (50%). Gefitinib treatment was well tolerated, with acne-like skin changes and rash occurring most frequently (12 patients, 38%). Symptomatic improvement was achieved in six patients (19%) and objective confirmed response in two (6%). Both responders were nonsmoking women (42 and 39 years) who experienced skin complications attributed to gefitinib. Response duration was 7 and 4+ months. Durable disease stabilisation (>6 months) was observed in one patient. The median survival was 7 (95% confidence interval [CI] 3–10) months and 1-year survival probability was 31% (95% CI 12–51%). Although objective response was rare, the response duration and symptomatic improvement following gefitinib treatment was of significant benefit. Responses achieved in young, nonsmoking women are intriguing but in keeping with previous observations. Further studies including clinical and biological prognostic factors are warranted.

## A case report of a non-small-cell lung cancer patient with brain metastases

**DM Kowalski^1^, K Zajda^1^ and M Krzakowski^1^**

*^1^M Sklodowska-Curie Memorial Cancer Centre, Warsaw, Poland*

A 58-year-old male patient was diagnosed with stage IV non-small-cell lung cancer (NSCLC) and brain metastases in May 2001. He had a Karnofsky performance status of 80%. Initial treatment comprised carboplatin and teniposide (four cycles) with whole-brain irradiation (30 Gy). Palliative radiotherapy was also given to the chest. The patient had a partial response, which was maintained for 16 months. Second-line cisplatin-based chemotherapy, given on disease progression, resulted in stable disease. He subsequently received oral gefitinib (‘Iressa’, ZD1839) 250 mg day^−1^ for 3 months as part of the ‘Iressa’ Expanded Access Programme, with disease stabilisation and improvement in quality of life and lifestyle. Gefitinib was well tolerated. Further chemotherapy has comprised carboplatin and gemcitabine. As of May 2003, the patient was still alive 24 months after diagnosis of brain metastases. This case illustrates that gefitinib is effective as third-line therapy for patients with metastatic NSCLC.

## Complete response of brain metastases from lung adenocarcinoma with gefitinib (‘Iressa’, ZD1839)

**P Maione^1^, L Musto^1^, A Rossi^1^, D Nicolella^1^, C Lombardi^1^ and C Gridelli^1^**

*^1^SG Moscati Hospital, Avellino, Italy*

In January 2001, a 51-year-old woman was diagnosed with lung adenocarcinoma and bone metastases. She had no smoking history or comorbidities. The patient was treated with six cycles of cisplatin+vinorelbine chemotherapy up to June 2001 and achieved a partial response. In December, she developed multiple brain metastases and was treated with palliative radiotherapy, which stabilised her brain disease. From January to June 2002, six cycles of gemcitabine were administered as second-line chemotherapy; however, the disease in her lungs and bone progressed. She was given palliative radiotherapy on thoracic (T8) vertebra, which improved her pain. In July 2002, the patient was referred to our hospital with an Eastern Cooperative Oncology Group performance status (PS) of 3, her serum level of carcinoembryonic antigen (CEA) was 1400 ng ml^−1^ and her epidermal growth factor receptor expression status was unknown. She was enrolled into the ‘Iressa’ Expanded Access Programme as an outpatient and commenced treatment with oral gefitinib (‘Iressa’, ZD1839) 250 mg day^−1^. In October 2002, a CT scan of her brain, chest and abdomen showed a complete response for brain metastases, minimal response for lung disease and stabilisation of bone disease. CEA serum level decreased to 104 ng ml^−1^ and both her symptoms and PS (1) improved. These data were confirmed in January 2003, when a further decrease of lung lesion tissue resulted in the patient achieving an overall partial response to gefitinib treatment. As of April 2003, the patient is continuing gefitinib and has not experienced any toxicity during treatment.

## Impact of third-line gefitinib (‘Iressa’, ZD1839) therapy on patients with advanced non-small-cell lung cancer who had failed prior platinum- and/or docetaxel-based regimens: case series

**A Mancuso^1^, O Martelli^1^, MR Migliorino^1^, R Di Salvia^1^ and F De Marinis^1^**

*^1^Forlanini Hospital, Rome, Italy*

Patients with non-small-cell lung cancer (NSCLC) pretreated with two to three standard chemotherapy regimens have a median overall survival time of 4 months (Massarelli *et al*. (2003) *Lung Cancer* **39**: 55). Gefitinib (‘Iressa’, ZD1839), an EGFR-TKI (epidermal growth factor receptor tyrosine kinase inhibitor), has recently shown favourable antitumour activity (30%) and symptom improvement (40%) in patients with NSCLC (Fukuoka *et al*. (2003) *J Clin Oncol* **21**: 2237–2246; Kris *et al*. (2002) *Proc Am Soc Clin Oncol* **21**: 292a). The efficacy of third-line gefitinib was analysed using tumour response rates, time to third progression (TTTP), time to death (TTD) and changes in Karnofsky performance status (PS). A total of 32 patients who had failed two previous chemotherapy regimens (median age 64 years; M/F, 69/31%; PS 1/2/3, 50/44/6%; locally advanced/metastatic disease, 69/31%) received 250 mg day^−1^ oral gefitinib. Disease control was observed in 20 out of 24 (83%) evaluable patients; two (8%) had partial remissions (both women with histologically confirmed adenocarcinoma) and 18 had stable disease. The median overall TTTP and TTD for 13 evaluable patients were 4 and 6 months, respectively. A positive correlation was observed between the related time to progression for second- and third-line treatment with docetaxel and gefitinib, respectively; 81% (13 out of 16) of the patients who progressed ⩾4 months after starting docetaxel had a TTTP of >4 months using gefitinib. PS improved rapidly (within 15 days) in 10 out of 32 patients (31%), allowing a reduction in analgesic use. Most drug-related adverse events were mild and reversible; treatment was discontinued in two patients (8%) with grade 2/3 diarrhoea and skin rash.

## Gefitinib (‘Iressa’, ZD1839) in heavily pretreated patients with metastatic non-small-cell lung cancer

**S Martín-Algarra^1^, A Gurpide^1^ and JM Lopez-Picazo^1^**

*^1^Servicio Oncologia Medica, Clinica Universitaria Navarra, Pamplona, Spain*

In October 2001, a 68-year-old woman was diagnosed with metastatic adenocarcinoma of the lung. From October 2001 to April 2002, the patient was treated with 4-week cycles of cisplatin, paclitaxel and gemcitabine, and achieved stable disease. However, 1 month after stopping treatment, an MRI scan showed a new brain lesion and bone metastases and a CT scan showed local progression. Treatment with oral gefitinib (‘Iressa’, ZD1839) 250 mg day^−1^ began in May 2002 as part of the ‘Iressa’ Expanded Access Programme. In June 2002, the patient underwent further imaging studies that showed a partial response in the brain and lung lesions. To date, 12 months after gefitinib treatment started, the patient has stable systemic disease, symptoms are improved and gefitinib continues to be well tolerated with no adverse events.

## Rapid symptom improvement in a patient with non-small-cell lung cancer given gefitinib (‘Iressa’, ZD1839)

**JL Martínez^1^**

*^1^Hospital Británico, Buenos Aires, Argentina*

A 60-year-old female patient who had smoked for 20 years was diagnosed in July 2002 with stage IV non-small-cell lung cancer with multiple brain metastases. Her performance status at diagnosis was 1. She received encephalic radiation and first-line paclitaxel/carboplatin, which achieved a best response of stable disease, but chemotherapy was discontinued due to a high level of toxicity. The patient started oral gefitinib (‘Iressa’, ZD1839) 250 mg day^−1^ on 5 March 2003, concomitantly with prednisolone and fenytoine, as part of the ‘Iressa’ Expanded Access Programme. Her only symptom, dry cough, disappeared after the first week of treatment. Performance status improved from 1 to 0 after 1 month. On 8 April 2003, an MRI scan showed stable disease and on 6 May 2003 a chest X-ray showed complete response in the primary lesion. To date, the patient is still receiving gefitinib without any adverse reaction, continues to have improved symptoms and quality of life, and is waiting for a new CT scan to confirm the complete response.

## Response and symptom improvement with gefitinib (‘Iressa’, ZD1839) 250 mg day^−1^ in a patient with poor performance status

**T Overbeck^1^ and F Griesinger^1^**

*^1^University of Göttingen, Göttingen, Germany*

A 62-year-old female patient with non-small-cell lung cancer (adenocarcinoma/bronchoalveolar carcinoma (BAC)) had previously undergone surgery and chemotherapy (four cycles of docetaxel/carboplatin+erythropoietin+granulocyte colony-stimulating factor). After 14 months without treatment, bronchoscopy showed an exophytic tumour with ∼50% stenosis of the right main bronchus. A chest X-ray (July 2002) revealed polytopic pulmonary nodules. The patient had a performance status (Eastern Cooperative Oncology Group) of 2–3. In July 2002, she began oral gefitinib (‘Iressa’, ZD1839) 250 mg day^−1^ in the ‘Iressa’ Expanded Access Programme. A chest X-ray in October 2002 revealed ∼50% size reduction of the multiple pulmonary nodules and a bronchoscopy showed endobronchial tumour regression. The dyspnoea reported before therapy improved after 4 weeks of gefitinib, oral steroids and terbutaline sulphate, with further improvement after 8 weeks. After 4 weeks of gefitinib the patient reported skin itching. In November 2002 the patient was hospitalised with sudden symptoms, including cough, dyspnoea and light fever, and vital capacity (VC) was reduced to ∼50%. Endoscopic signs were similar to acute bronchitis. Symptoms improved with antibiotics/prednisolone and gefitinib was discontinued for ∼1 month. The episode was interpreted as interstitial pneumonia (IP) due to medication with gefitinib. In January 2003 (after resolution of clinical signs of IP, VC ∼82%) gefitinib and corticosteroids were restarted because of progressive BAC. Therapy was accompanied by nausea, vomiting, fatigue and mucositis. Again gefitinib improved dyspnoea. In April 2003 she presented with deep vein thrombosis. This patient had a significant and subjective response to gefitinib. The clinical course was complicated by IP, which was successfully treated. Rechallenge of the tumour by gefitinib resulted in significant symptom improvement.

## Gefitinib (‘Iressa’, ZD1839) produces disease control and significant improvement in symptoms in patients with advanced non-small-cell lung cancer

**AG Pallis^1^, D Mavroudis^1^, N Androulakis^1^, J Souglakos^1^, C Kourousis^1^, V Bozionelou^1^, N Vardakis^1^, IG Vlachonikolis^2^ and V Georgoulias^2^**

*^1^University Hospital of Heraklion, Greece; ^2^School of Medicine, University of Crete, Crete, Greece*

A total of 31 patients (M/F, 27/4; median age 60 (range 43–65) years) with advanced unresectable, progressive non-small-cell lung cancer (NSCLC) (stage IIIb, 15 patients; stage IV, 16 patients), who had been treated with ⩾2 prior chemotherapy regimens, received gefitinib (‘Iressa’, ZD1839) orally, 250 mg day^−1^ for a median duration of 8 (range 3–32) weeks. Four patients were WHO performance status (PS) 0, 21 were PS 1 and 6 were PS 2. One patient achieved partial response and nine patients had stable disease, producing a disease control rate of 10 out of 31 (32%). After a median follow-up of 18 weeks, 12 patients were still alive. Median overall survival (OS) was 23 (range 4–40) weeks, median duration of response was 11 (range 1–32) weeks and median time to progression (TTP) was 9 (range 3–31) weeks. There was no difference in OS according to histological type (*P*=0.4169), disease stage (*P*=0.496) or number of prior treatments (*P*=0.979). There was a significant difference for TTP between patients with PS 0–1 and those with PS 2 (11 (range 3–31+) weeks *vs* 4 (range 3–8) weeks; *P*<0.001) and for OS (median survival: 27 weeks *vs* 6 weeks; *P*<0.001). Symptoms were significantly improved in 39% of patients after 6 weeks compared with baseline (*P*<0.001). Median time to symptom improvement was 3 (range 2–4) weeks. The majority of adverse events were grade 1/2 and included skin rash (58%), diarrhoea (28%) and nausea/vomiting (19%). In these heavily pretreated patients with advanced NSCLC, gefitinib was well tolerated and produced disease control and symptom improvement.

## Symptom improvement following gefitinib (‘Iressa’, ZD1839) therapy in a pretreated patient with non-small-cell lung cancer

**N Pavlakis^1^**

*^1^Royal North Shore Hospital, St Leonards, New South Wales, Australia*

In November 2001, a 42-year-old male smoker was diagnosed with non-small-cell lung cancer following a 3-month history of cough, weight loss and increasing shortness of breath. A CT scan revealed a mass in the left lung and multiple pulmonary nodules. In addition, malignant cells were detected in the left supraclavicular lymph node by fine needle aspiration biopsy. In December 2001, the patient received combination chemotherapy (cisplatin and gemcitabine) but presented with progressive disease after two cycles. In February 2002 the patient was treated with docetaxel but after six cycles disease progressed in the chest and spleen. Cerebral metastases were diagnosed in June 2002, and treated with radiation therapy (20 Gy in five fractions). Following commencement of gefitinib (‘Iressa’, ZD1839) 250 mg day^−1^, in December 2002, dramatic symptom improvement was observed, including improved performance status to 1. Furthermore, extra-cranial disease was stable, although progression of cerebral lesions occurred. Gefitinib was generally well tolerated; however, the patient did report a facial skin rash. The patient continued to receive gefitinib therapy during neurosurgical analysis of these symptomatic lesions. However, gefitinib therapy was terminated in February 2003 when the patient was diagnosed with progressive disease of the central nervous system, and developed hydrocephalus and meningeal disease. In conclusion, gefitinib treatment was effective in alleviating symptoms in this patient with brain metastases.

## An elderly patient with extensive non-small-cell lung cancer was effectively treated with first-line gefitinib (‘Iressa’, ZD1839) followed by combined chemotherapy

**M Pesek^1^ and B Eliasova^1^**

*^1^Faculty Hospital, Plzen, Czech Republic*

A 70-year-old nonsmoking female patient suffering from cough, chest pain, fever and severe comorbidity was diagnosed with extensive stage IV pulmonary adenocarcinoma after clinical investigation and video-assisted thoracoscopic surgery. The disease had metastasised into both lungs, mediastinal lymph nodes and vertebral column, and a left-sided malignant pleural effusion was found. The patient refused the proposed combined chemotherapy due to anxiety. She received oral gefitinib (‘Iressa’, ZD1839) 250 mg day^−1^ from 5 January to 19 March 2002. The best response was stable disease lasting for 2 months, accompanied by remission of pleural effusion and marked improvement of cough, dyspnoea and chest pain, which occurred within 2 weeks of starting treatment. There were some side effects, particularly nausea and diarrhoea which resolved with the help of standard pharmacological therapy. After minimal radiological progression, the patient agreed to combined chemotherapy. There were four cycles of combination therapy, consisting of gemcitabine (650 mg m^−2^; Days 1 and 8), paclitaxel (65 mg m^−2^; Days 1, 8 and 15) and carboplatin (AUC 2; Days 1, 8 and 15). Cycles were repeated from Day 35. This therapy resulted in partial tumour remission, documented by chest X-ray and Tc-99 m Sestamibi gammagraphy. Symptomatic improvement also occurred. More than 18 months from the date of diagnosis, the patient is still alive, with slowly progressing malignant disease. This case report documents the feasibility of first-line gefitinib treatment in an elderly patient with severe comorbidity. Moreover, subsequent combined chemotherapy resulted in partial remission of the tumour.

## Efficacy, tolerability and improvement of symptoms in heavily pretreated patients with locally advanced or metastatic non-small-cell lung cancer treated with gefitinib (‘Iressa’, ZD1839)

**V Petersen^1^, P Grau^1^, F Schneller^1^ and C Peschel^1^**

*^1^3 Medizinische Klinik, Klinikum rechts der Isar, Munich, Germany*

A total of 33 patients (30 patients with metastatic disease, three patients with locally advanced disease; median age 62 years) with non-small-cell lung cancer (NSCLC) previously treated with chemotherapy received oral gefitinib (‘Iressa’, ZD1839) 250 mg day^−1^. Gefitinib was given as second-line treatment in nine patients, third line in 16 patients, fourth line in five patients and fifth line in three patients. Seven patients developed stable disease (⩾24 weeks in two patients) and one patient had a partial response. There was no correlation between response to chemotherapy and response to gefitinib, although the two patients who developed stable disease for ⩾24 weeks received gefitinib as third-line therapy. In all, 11 patients reported an improvement in symptoms after 1 week of treatment. Improvements in quality of life, six patients; cough, three patients; pain, one patient; and dyspnoea, two patients were observed. One patient reported an improvement in appetite and an increase in body weight. However, in the majority of patients, symptom control stopped after 6 weeks of treatment. Ten patients had no side effects with gefitinib. In total, 16 patients developed acne (grade 1/2, 14 patients; grade 3, one patient; grade 4, one patient), seven patients developed dry skin, seven patients reported diarrhoea (grade 1, six patients; grade 3, one patient) and one patient developed hypertrichosis. In this heavily pretreated population with locally advanced or metastatic NSCLC, gefitinib was effective and produced rapid reduction in symptoms in over one-third of patients.

## Efficacy of gefitinib (‘Iressa’, ZD1839) in a patient with advanced non-small-cell lung cancer with metastatic spread to the brain

**L Petruzelka^1^ and M Zemanova^1^ [a]**

*^1^University Hospital, Charles University, Prague, Czech Republic*

A 61-year-old female nonsmoker, who suffered from coronary artery disease and chronic obstructive pulmonary disease, initially presented with coughing, dyspnoea and weight loss. After she developed superior vena cava syndrome in December 1998, examination revealed squamous-cell carcinoma of the right upper lobe with mediastinal involvement and mediastinal lymph node enlargement (stage IIIb). Between January and May 1999, four cycles of cisplatin and vinorelbine were administered, plus concurrent radiation with cycles 2–4. The patient had a partial response; however, tolerability was poor, with febrile neutropenia and nephrotoxicity. The first relapse occurred in April 2000, consisting of a brain lesion (adenocarcinoma), which was resected. Despite whole-brain irradiation in June 2000, a further lesion occurred in another location, which was resected in November, with surgery performed in September 2001. In May 2002, a chest X-ray showed disease progression and the patient complained of cough, dyspnoea and weight loss. Owing to her poor tolerability of previous chemotherapy and comorbid conditions, oral gefitinib (‘Iressa’, ZD1839) 250 mg day^−1^ therapy began in June 2002. A partial response was observed in August and symptoms improved. Gefitinib was generally well tolerated with minimal toxicity (mild acneiform dermatitis and sporadic episodes of diarrhoea). In February 2003, the patient had stable disease and Eastern Cooperative Oncology Group performance status 1. Gefitinib therapy was durable and effective in preventing local recurrence following radical chemoradiotherapy with no disease progression after 9 months of treatment. Furthermore, 18 months after the last surgery, there has been no disease recurrence in the brain.

## Regression of metastatic non-small-cell lung cancer in a chemonaive patient treated with gefitinib (‘Iressa’, ZD1839)

**L Petruzelka^1^ and M Zemanova^1^ [b]**

*^1^University Hospital, Charles University, Prague, Czech Republic*

This case report describes the treatment of a 64-year-old male exsmoker who was diagnosed with squamous-cell carcinoma in February 2002, although a nodule in the right supraclavicular area had been palpable for several months. This patient also had hypertension, obesity, diabetes mellitus, dyslipoproteinaemia and chronic obstructive pulmonary disease. Surgical resection was performed in February 2002, including nonradical removal of the supraclavicular lymph nodes. Staging procedures identified a possible primary tumour in the right upper lobe, in addition to the supraclavicular involvement, and multiple metastatic bone lesions (stage IV, T1N3M1). At this time the patient was asymptomatic and refused chemotherapy; however, radiotherapy was delivered to the supraclavicular area from 22 April–15 May 2002. As part of the ‘Iressa’ Expanded Access Programme, treatment with oral gefitinib (‘Iressa’, ZD1839) 250 mg day^−1^ commenced on 8 July 2002, and a partial response was observed on 26 August 2002 and was confirmed on 4 October 2002. At the last visit (11 April 2003), this response was sustained and there was no recurrence in the right supraclavicular area. Regression of the bone metastases was also seen, with a bone scan in January 2003 showing no metastatic lesions in the ribs. Quality of life improved after 2 months and the treatment was well tolerated, with mild skin rash (grade 1) and conjunctivitis (grade 1/2), manageable with routine supportive care. Gefitinib therapy has demonstrated efficacy in this chemonaive patient, resulting in regression of both the primary tumour and bone metastases. This patient has now received gefitinib for 9 months and treatment is ongoing.

## Gefitinib (‘Iressa’, ZD1839) as treatment for non-small-cell lung cancer

**E Razis^1^, S Papadopoulos^1^, D Skarlos^1^, M Exarchakos^1^, C Christodoulou^1^, M Karina^1^, M Xylouri^1^ and S Labropoulos^1^**

*^1^Hygeia Hospital, Athens, Greece*

A total of 53 patients with non-small-cell lung cancer (NSCLC) commenced therapy with gefitinib (‘Iressa’, ZD1839) 250 mg day^−1^ between March 2001 and November 2002 as part of the ‘Iressa’ Expanded Access Programme. Three patients received gefitinib as first-line treatment, 16 as second-line, 17 as third-line, 11 as fourth-line and 6 as >fourth-line. One patient had a very good partial response to gefitinib, two patients were stable for 13.3 and 2.7+ months and 39 patients progressed during therapy. Nine patients are still on treatment after <1–5.5 months. The median duration of treatment for the whole group was 2.85 (range <1–13.3) months. With median follow-up 8.8 months, 29 patients have died from disease progression and 1 from acute respiratory distress syndrome and disseminated intravascular coagulation. Time to disease progression was 3.5 months for patients being treated with first- or second-line gefitinib, 2.4 months for those receiving third-line gefitinib and 2.9 months for those receiving ⩾fourth-line therapy. Median survival after initiation of gefitinib was 4.6 months. Six patients reported grade 3 toxicities (rash, four patients; diarrhoea, one patient; congestive heart failure, one patient), and one patient experienced grade 4 diarrhoea. All toxicities resolved after discontinuing gefitinib. Tumour epidermal growth factor receptor expression determined by immunohistochemistry in 24 patients (11 positive, 13 negative) did not correlate statistically with time to progression or survival, although the numbers are too small to make definite conclusions. However, some patients with advanced NSCLC benefit from gefitinib, as demonstrated by a longer time to progression compared with historical data.

## Gefitinib (‘Iressa’, ZD1839) in relapsed non-small-cell lung cancer

**M Reck^1^ and U Gatzemeier^1^**

*^1^Krankenhaus Großhansdorf, Großhansdorf, Germany*

A total of 40 patients (12 female and 28 male) with relapsed non-small-cell lung cancer, received gefitinib (‘Iressa’, ZD1839) between March 2002 and April 2003. The median age was 58 (range 40–80) years. Four patients showed evidence of activity with gefitinib. Patient A, a female with metastatic adenocarcinoma (T2N2M1), had previously received radiotherapy and six cycles of paclitaxel/gemcitabine. Gefitinib treatment duration was 6 months. She demonstrated a minor tumour response and the worst toxicity was grade 1 pruritus. Patient B, a male with bronchioloalveolar carcinoma (M1), had previously undergone resection of the left lower lobe and had received two cycles of carboplatin/vinorelbine and four cycles of gemcitabine. Gefitinib treatment was maintained for 9 months with a best response of stable disease. The patient reported grade 1 exanthema and diarrhoea. Patient C, a male with squamous-cell carcinoma (T3N3M0), had previously received three cycles of carboplatin/paclitaxel and radiotherapy. Duration of gefitinib treatment was 6.5 months with a best response of stable disease. The worst toxicity was grade 2 exanthema. Patient D, a female patient with adenocarcinoma (T4N3M0), had previously received six cycles of carboplatin/paclitaxel plus *N*-acetyldinaline (CI-994)/placebo, two cycles of Bay 38–3441 (an angiogenesis inhibitor) and two cycles of gemcitabine. Gefitinib treatment resulted in stable disease with no toxicity and was continued for 7 months. Despite heavy pretreatment with chemotherapy and radiation therapy gefitinib produced tumour control over a remarkable period in four out of 40 patients, which was combined with symptom relief. Treatment with gefitinib was straightforward, feasible in an outpatient setting and was well tolerated.

## Good response in primary tumour, brain, cerebellar and liver metastases after 2 months’ gefitinib (‘Iressa’, ZD1839) treatment

**ER Roggero^1^, G Busi^1^ and A Pedrazzini^1^**

*^1^Humaine Clinica, Locarno, Switzerland*

A female patient aged 79 years was first diagnosed in January 2001 with non-small-cell lung cancer, with metastases in the liver and contralateral lung. The patient was highly symptomatic (persistent cough, asthenia and dyspnoea at rest) and received two different chemotherapy regimens, each for 1 month (vinorelbine/cyclophosphamide then irinotecan/gemcitabine). However, her general condition deteriorated and she complained of headaches. Tumour progression was confirmed in the lung and the liver. The patient then started oral gefitinib (‘Iressa’, ZD1839) 250 mg day^−1^ in April 2002, as part of the ‘Iressa’ Expanded Access Programme. At this time, multiple lesions were documented in the lung, liver, peritoneum, mediastinum and bones. In addition, a CT scan showed multiple diffuse brain and cerebellar metastases. At this time, the patient had a performance status (WHO) of 2. After 2 months’ gefitinib treatment, significant reductions in the primary tumour and brain metastases were documented on chest X-ray and CT scan. The patient's symptoms of fatigue, cough and pain had improved, and she had a good performance status lasting for approximately 7 weeks. Although the patient developed herpes zoster during gefitinib therapy, this responded well to a 10-day course of acyclovir and a 6-day interruption of gefitinib. Gefitinib treatment continued for 6 months until the patient's death in September 2002 due to rapid progression of liver metastases.

## Activity of the epidermal growth factor receptor inhibitor gefitinib (‘Iressa’, ZD1839) in refractory non-small-cell lung cancer

**H Soto Parra^1^, R Cavina^1^, P Zucali^1^, E Campagnoli^1^, F Latteri^1^, G Biancofiore^1^, G Abbadessa^1^, E Morenghi^1^ and A Santoro^1^ [a]**

*^1^Istituto Clinico Humanitas, Rozzano, Italy*

The antitumour activity and tolerability of oral gefitinib (‘Iressa’, ZD1839) 250 mg day^−1^ were assessed in a series of patients with previously treated, advanced non-small-cell lung cancer (NSCLC), as part of the ‘Iressa’ Expanded Access Programme. To be eligible, patients were required to have histologically or cytologically proven advanced or metastatic NSCLC, prior chemotherapy with at least one cisplatin-containing chemotherapy regimen or contraindication to cytotoxic drugs, Eastern Cooperative Oncology Group performance status ⩽2, and adequate haematological, renal and hepatic parameters. Although gefitinib was supplied on a named-patient basis, all patients provided signed, informed consent. Patient re-evaluation was performed every 4–6 weeks. A total of 73 consecutive patients were enrolled, having been diagnosed with NSCLC from February 2001 to April 2003. Nonhaematological toxicity was generally mild: there was grade 3 skin rash and grade 3 diarrhoea in 5 and 1% of cases, respectively. Response rate, including complete and partial response, was 10%; an additional 44% of patients achieved stable disease for an overall disease control of 54%. Median survival for all patients was 4 months, reaching 6 months for patients with disease control. Gefitinib has promising activity with a good toxicity profile in patients with progressive NSCLC who have received one or two prior chemotherapy regimens. Currently, the development of epidermal growth factor receptor tyrosine kinase inhibitors represents the most appealing biological approach for NSCLC. More studies are needed.

## Gefitinib (‘Iressa’, ZD1839) in elderly patients with progressive pretreated non-small-cell lung cancer: results from the Istituto Clinico Humanitas

**H Soto Parra^1^, R Cavina^1^, P Zucali^1^, E Campagnoli^1^, F Latteri^1^, G Biancofiore^1^, G Abbadessa^1^, E Morenghi^1^ and A Santoro^1^ [b]**

*^1^Istituto Clinico Humanitas, Rozzano, Italy*

The antitumour activity and tolerability of gefitinib (‘Iressa’, ZD1839) were evaluated in a series of elderly patients (aged ⩾70 years) with advanced non-small-cell lung cancer (NSCLC), as part of the ‘Iressa’ Expanded Access Programme. From February 2001 to the present, 140 patients with advanced/metastatic NSCLC, who had received prior chemotherapy or had a contraindication to cytotoxic drugs, and had an Eastern Cooperative Oncology Group performance status (PS) ⩽2, were treated with oral gefitinib 250 mg day^−1^. From this group, 31 elderly patients were evaluated: male/female 27/4; PS ⩽1, 21; median age 74 (range 70–82) years. A total of 20 patients had received prior chemotherapy (one regimen, 16 patients; two regimens, four patients), including 15 who had received platinum-based chemotherapy. In all, 11 patients had received no previous treatment due to medical contraindications. No objective response was observed. In total, 18 patients had disease stabilisation (58%) for a median duration of 5.5 (range 1–14) months. Gefitinib was well tolerated; the most frequent toxicities were grade 1/2 and 3 skin rash in 42 and 10% of cases, respectively, and grade 1/2 and 3 diarrhoea in 29 and 3%, respectively. Median survival for all elderly patients and those with stable disease was 3 and 5 months, respectively, and 1-year survival was 22 and 28%, respectively. This series shows that gefitinib may achieve disease control in >50% of elderly patients with progressive NSCLC after one or two chemotherapy regimens, with an excellent toxicity profile. Gefitinib could be a new therapeutic option for elderly patients with medical contraindications to standard chemotherapy.

## Analysis of responses to gefitinib (‘Iressa’, ZD1839) according to epidermal growth factor receptor expressed as staining intensity or percentage of immunoreactive cells

**H Soto Parra^1^, A Santoro^1^, R Cavina^1^, F Latteri^1^, P Zucali^1^, E Campagnoli^1^ and M Roncalli^1^ [c]**

*^1^Istituto Clinico Humanitas, Rozzano, Italy*

Of 140 patients with advanced non-small-cell lung cancer treated with oral gefitinib (‘Iressa’, ZD1839) 250 mg day^−1^ as part of the ‘Iressa’ Expanded Access Programme, 36 patients (aged 37–60 years; male : female, 28 : 8; performance status ⩽1 in 28 patients; 31 patients previously treated) had paraffin-embedded tissue available. One patient experienced complete remission, three had partial remission, 16 had stable disease and 16 progressed (disease control rate 55.6%). The median survival was 4 months and 1-year survival was 12.3%. Epidermal growth factor receptor (EGFR) was evaluated by immunohistochemistry using the monoclonal antibody EGFRAb-10 (clone 111.6, Neomarkers) diluted 1 : 100 and the En Vision detection system (Dako). The percentage of neoplastic cells showing membrane immunoreactivity (IR) was semiquantitatively evaluated (range 0–100%). Response to gefitinib according to EGFR expressed as staining intensity or percentage of IR cells was (Table 1

**Table 1:** 

	**Patients, *n***	**Responses, *n* (%)**	**Disease control, *n* (%)**	**Progression, *n* (%)**
*Staining intensity*				
2+/3+	16	4 (25)	8 (50)	8 (50)
0/1+	20	—	12 (60)	8 (40)
				
*IR cells*				
HE	15	3 (20)	7 (47)	8 (53)
NLE	21	1 (5)	13 (62)	8 (38)

2+/3+=medium/strong; 0/1+=negative/faint staining intensity; HE=high expressors (⩾20% IR cells); NLE=negative/low expressors (0–19% IR cells).

).

Overall, 44.4 and 41.7% of patients had staining intensity 2+/3+ and HE, respectively. According to these results no relationship has been documented between EGFR expression and disease control. However, almost all responses were observed in HE patients or those with medium/strong immunoreactivity. These issues deserve further clinical and biological evaluation.

## Long-term responders to gefitinib (‘Iressa’, ZD1839): maintenance of tolerability, and propensity for central nervous system failure

**BN Stein^1^, D Kotasek^1^, FX Parnis^1^ and C Bampton^1^**

*^1^Ashford Cancer Centre, Ashford, Australia*

Four patients with metastatic non-small-cell lung cancer were evaluated as a case series. Characteristics of the patients included: male : female, 3 : 1; age range, 46–76 years; performance status (PS), 1; metastases to lung or bone; and exsmokers, 2. All four patients had previously been treated with carboplatin/gemcitabine and two had also received weekly docetaxel. One patient had received palliative radiation to the bone. One patient had a partial response and three patients had stable disease with previous therapy. Following treatment with oral gefitinib (‘Iressa’, ZD1839) 250 mg day^−1^, all patients had a partial response and treatment was continued for 6–9 months. The responses were clinically meaningful, which was demonstrated in one patient by an improvement in PS from 2 (dependent on oxygen) to 0 (no longer dependent on oxygen). Two patients discontinued concomitant opiate therapy. In addition to effects on quality of life and lifestyle, improvement in pain was experienced within days and improvement in dyspnoea within weeks. Toxicity was manageable: all four patients had grade 1 skin rash and two patients had grade 1 diarrhoea, and there was no evidence of cumulative toxicity during the period of treatment. Two patients progressed, both in the meninges (one also with small volume multifocal central nervous system disease); however, the primary disease remained controlled.

## Long-term disease stabilisation with gefitinib (‘Iressa’, ZD1839) treatment in a patient with advanced non-small-cell lung cancer and multiple metastatic sites

**R van der Kamp^1^, MGJ Koolen^1^ and HM Jansen^1^**

*^1^Amsterdam, The Netherlands*

In July 2002, a 56-year-old female ex-smoker presented with symptoms of dysarthria, paraesthesia, decreased right-leg strength, pain in the right side of the thorax, dyspnoea and a dry cough, which had all appeared 2 months previously. A CT scan revealed a tumour that had invaded the mediastinum, with multiple pathological lymph nodes and multiple metastases in the lung. Multiple brain metastases were also observed by MRI scan. The disease was histologically confirmed as stage IV non-small-cell lung cancer (NSCLC). Owing to the relatively good physical condition of the patient (Eastern Cooperative Oncology Group performance status 1) and the low chance of success of chemotherapy due to the brain metastases, initial treatment consisted of radiotherapy of the brain (5 × 4 Gy) in July 2002. Treatment with oral gefitinib (‘Iressa’, ZD1839) 250 mg day^−1^ was initiated in August 2002. In September 2002 the patient had a mild skin rash and nausea. Celecoxib had also been used for some weeks and, once the dose of this comedication was decreased, the skin rash disappeared. In September and October 2002, the patient's walking ability seemed to be becoming progressively impaired; however, the deterioration was mild and the condition has since stabilised. At her most recent visit, in March 2003, the patient's overall condition was stable and gefitinib treatment is ongoing. This patient with advanced NSCLC, including multiple brain metastases, has experienced disease stabilisation for more than 9 months while on gefitinib treatment. This is an unexpectedly long period of stable disease, to which gefitinib may have contributed.

## Long-term response induced by gefitinib (‘Iressa’, ZD1839) in a patient with bronchioalveolar cell carcinoma

**N van Zandwijk^1^ [a]**

*^1^The Netherlands Cancer Institute, Amsterdam, The Netherlands*

A 65-year-old female nonsmoker was diagnosed with bronchioalveolar carcinoma (BAC) in 1997 and underwent left-lung pneumectomy. In March 1999, disease recurrence (stage IV) was identified with a slowly progressing invasion in the other lung. The patient was treated with 1 dose of cisplatin/gemcitabine followed by gemcitabine alone from February to October 2000, which resulted in stable disease. However, in October the patient's condition deteriorated, with a considerable decline in lung function, reducing her performance status (PS) (WHO) to 2–3, and so prednisone therapy was initiated. Treatment with oral gefitinib (‘Iressa’, ZD1839) began in July 2001, leading to a gradual improvement of pulmonary symptoms over 3 months, with prednisone reduced and finally discontinued in June 2002. An X-ray revealed a gradual clearing of the right lung in January 2002, meeting the criteria for a partial response, and the patient's PS had improved to 1. The patient initially experienced mild diarrhoea, and also reported intermittent skin rashes. Gefitinib has provided a long-term response for this patient with stage IV non-small-cell lung cancer. Treatment is ongoing after more than 9 months and the partial response has been sustained. Significant quality-of-life improvements have been observed, and the patient's PS is minimally impaired despite pulmonary infections. This case report demonstrates that a relatively slow onset of response to therapy may be seen with BAC patients. In addition, patients with severely impaired pulmonary function may show a favourable response to epidermal growth factor receptor inhibitors, such as gefitinib.

## Quality-of-life benefits for a patient with non-small-cell lung cancer and brain metastases taking gefitinib (‘Iressa’, ZD1839)

**N van Zandwijk^1^ [b]**

*^1^The Netherlands Cancer Institute, Amsterdam, The Netherlands*

Stage IV non-small-cell lung cancer with lymphangitic spread was diagnosed in a 55-year-old male ex-smoker with no significant medical history in October 1999. He was treated with cisplatin and gemcitabine for 3 months followed by gemcitabine alone for 4 months, and a partial response was achieved. However, the disease recurred in September 2000 and oral paclitaxel was given until disease progression in June 2001. Treatment with gefitinib (‘Iressa’, ZD1839) began in July 2001. After an initial improvement, pleural fluid increased and evacuation and subsequent pleurodesis were performed. Thereafter, a gradual symptomatic improvement was observed and the patient resumed work in January 2002. His performance status (WHO) was 0–1. In September 2002, impaired coordination indicated the development of brain metastases, with examination revealing approximately 20 small lesions. A chest X-ray showed stable disease. Radiotherapy to the brain (10 × 3 Gy) was administered in October 2002 and gefitinib treatment continued. Symptom improvement occurred gradually and the disease stabilised. The patient resumed work (60%) again in May 2003, with a good general condition and WHO performance status 1–2. In this case, gefitinib produced disease stabilisation and excellent quality-of-life benefits, with the patient able to return to work despite advanced metastatic disease. Combined radiotherapy and gefitinib treatment were well tolerated and resulted in a durable clinical response, which has currently lasted for more than 6 months. Gefitinib did not protect against brain metastases; however, the treatment of brain metastases during gefitinib therapy may be worthwhile.

## Disease stabilisation following gefitinib (‘Iressa’, ZD1839) therapy in a patient with metastatic non-small-cell lung cancer and very poor performance status

**MD Vincent^1^**

*^1^London Regional Cancer Centre, London, Canada*

In January 2002, a 56-year-old male smoker presented with non-small-cell lung cancer metastases to the ipsilateral lung and pleura, and an Eastern Cooperative Oncology Group performance status (PS) of 1. The patient had been exposed to asbestos in 1974 although no related lung complications were present. Four cycles of carboplatin plus paclitaxel were administered. The patient experienced a partial response to this treatment regimen but had a bad allergic reaction to paclitaxel that required in-patient administration. He had also become very emaciated and hypercalcaemic, and his PS had deteriorated to 4. Oral gefitinib (‘Iressa’, ZD1839) 250 mg day^−1^ was administered with concomitant narcotics, megace and oxygen. Following initiation of gefitinib the patient experienced a 2-month period of disease stabilisation and symptom control, for which he was grateful. Minor improvements in pain, dyspnoea and quality of life were evident within 1 month of starting gefitinib, and the patient's serum alkaline phosphatase declined from 201 to 140 IU L^−1^ during this time. Gefitinib was well tolerated with no side effects. Although the patient died after 3 months of treatment, it is probable that gefitinib therapy held his disease in check for 2 months, when he would otherwise have continued to decline rapidly. Moreover, it is speculated that earlier initiation of gefitinib may have stabilised the patient's disease when his PS had been better.

